# GammaGAN: Gamma-Scaled Class Embeddings for Conditional Video Generation

**DOI:** 10.3390/s23198103

**Published:** 2023-09-27

**Authors:** Minjae Kang, Yong Seok Heo

**Affiliations:** 1 Department of Electrical and Computer Engineering, Ajou University, Suwon 16499, Republic of Korea; dreamer29@ajou.ac.kr; 2 Department of Artificial Intelligence, Ajou University, Suwon 16499, Republic of Korea

**Keywords:** class embeddings, conditional generative adversarial networks, conditional video generation, GammaGAN, generative adversarial networks, projection discriminator, video generation

## Abstract

In this paper, we propose a new model for conditional video generation (GammaGAN). Generally, it is challenging to generate a plausible video from a single image with a class label as a condition. Traditional methods based on conditional generative adversarial networks (cGANs) often encounter difficulties in effectively utilizing a class label, typically by concatenating a class label to the input or hidden layer. In contrast, the proposed GammaGAN adopts the projection method to effectively utilize a class label and proposes scaling class embeddings and normalizing outputs. Concretely, our proposed architecture consists of two streams: a class embedding stream and a data stream. In the class embedding stream, class embeddings are scaled to effectively emphasize class-specific differences. Meanwhile, the outputs in the data stream are normalized. Our normalization technique balances the outputs of both streams, ensuring a balance between the importance of feature vectors and class embeddings during training. This results in enhanced video quality. We evaluated the proposed method using the MUG facial expression dataset, which consists of six facial expressions. Compared with the prior conditional video generation model, ImaGINator, our model yielded relative improvements of 1.61%, 1.66%, and 0.36% in terms of PSNR, SSIM, and LPIPS, respectively. These results suggest potential for further advancements in conditional video generation.

## 1. Introduction

Deep learning models currently dominate video generation tasks because they can create high-quality and realistic videos. The challenge of conditional video generation, specifically the generation of plausible videos from a single image with a class label, has prompted intensive research. Various deep learning methods using generative adversarial networks (GANs) [1], transformer models [2,3,4,5], and diffusion models [6,7,8,9] have been extensively explored. Although transformer and diffusion models have outperformed GANs in certain aspects [9,10], GANs are efficient in video generation due to their simplicity and relatively shorter inference time. However, traditional video generation methods using conditional generative adversarial networks (cGANs) encounter difficulties in effectively utilizing the class information as a condition. Typically, these methods concatenate class labels to feature maps in a generator and a discriminator through simple concatenation [11], which makes it difficult to utilize conditional information [12]. In particular, the role of the discriminator is to distinguish between the distribution of the generator and that of real data on the set of data samples and conditional class labels. The performance of the discriminator is crucial for improving generative quality and stability of the cGANs.

Given these considerations, increased research has been conducted on methods for providing class labels to discriminators. Techniques for utilizing class information in discriminators can generally be categorized into two types: injecting a class label directly and employing auxiliary classifiers. The former is commonly achieved by concatenating [11,13,14,15,16,17,18] or by projecting the class information using class embeddings [12,19,20,21]. On the other hand, auxiliary classifiers [22,23,24,25,26] aim to use class information effectively through classification.

As an alternative to the concatenation method for providing the conditional class information, the projection discriminator [12] can be applied to video generation [27], which offers the benefit of learning the relationship between feature vectors and class embeddings by applying the inner product between them. However, when a projection discriminator is employed for video generation, it tends to diminish class differences, resulting in similar results despite different classes. This is because the projection discriminator is vulnerable to overfitting and mode collapse [28,29].

**Figure 1:**
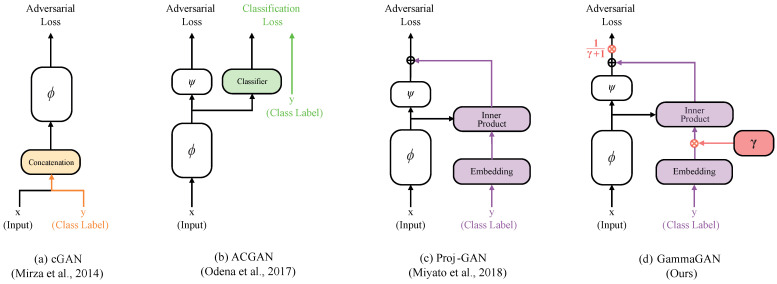
Various class label utilization methods of cGANs. (**a**) cGAN [11] uses the concatenation method (orange), and (**b**) ACGAN [22] employs classification loss and a classifier (green) to utilize conditional information. (**c**) Proj-GAN [12] and (**d**) GammaGAN use the projection method (purple) with class embeddings. In GammaGAN (our proposed method), we define a network consisting of two streams: the data stream (**left**) and the class embedding stream (**right**). The class embeddings are scaled by γ (red) to emphasize class conditional information, and outputs are normalized by $\frac{1}{\gamma +1}$ (red) to balance the outputs.

To address the limitations of current projection discriminators in video generation, in this paper, we propose a novel technique, GammaGAN, to amplify the differences between classes and improve the quality of videos. This is achieved by adaptively scaling the class embeddings and normalizing the outputs, as shown in Figure 1. Scaled class embeddings emphasize class information, and normalized outputs automatically learn to effectively balance the feature vectors and class embeddings. Consequently, our discriminator can provide proper feedback to the generator during training by effectively distinguishing class differences, which results in enhanced video quality. Our experiments on the MUG facial expression dataset [30] demonstrated the effectiveness of our approach, with quantitative and qualitative analyses showing improved quality compared to prior video generation models such as VGAN [31], MoCoGAN [32], and ImaGINator [33]. In particular, our approach led to relative improvements of 1.61%, 1.66%, and 0.36% in terms of PSNR, SSIM, and LPIPS, respectively, on the MUG dataset compared to ImaGINator [33].

To provide an overview, our main contributions are summarized as follows:

- We propose GammaGAN, an enhanced video discriminator network for conditional video generation that incorporates two novel techniques: scaling class embeddings and normalizing outputs.
- Scaled class embeddings emphasize class conditional information, thereby enhancing the distinction between different classes.
- Our technique for normalizing outputs balances the outputs of the model. This enables the prioritization between feature vectors and class embeddings during training, leading to improved video quality.

## 2. Related Work

### 2.1. Video Generation

The goal of video generation is to produce realistic videos. Video generation is a challenging problem due to the high dimensionality of video data and the difficulties in creating effective feature vectors [34]. Various generative models have recently been proposed for video generation. Generative adversarial networks (GANs), initially introduced in the image domain [1,14,35,36,37,38,39,40,41,42], have been adapted for the video domain [27,31,32,43,44]. In addition, advanced models, such as diffusion models [45,46] and transformer models [47,48,49], have been introduced for video generation tasks. These models have demonstrated impressive performance, often surpassing the results achieved by traditional GANs [9,10]. However, these advanced models incur higher computational costs and longer inference times.

### 2.2. Conditional Generative Adversarial Networks

Generative adversarial networks (GANs) are models designed to produce realistic images. Conditional generative adversarial networks (cGANs) allow us to provide conditional information to a model during training using a class label. In the early stages of cGANs research, class conditional information was provided to a model by concatenation [11]. This is typically done by directly injecting it into the input [13] or by injecting it into a hidden layer [14,15,16,17,18]. However, projection-based methods [12,19,20,21] have been developed to effectively utilize class information for cGANs. This method involves generating two types of embeddings (feature vectors and class embeddings) and then calculating the inner products between them to measure their similarities. Nevertheless, when the projection method is applied to the task of video generation [27], it tends to decrease class differences during training because it still suffers from overfitting and mode collapse [28,29]. To address this problem, we propose a novel technique that focuses on enhancing class differences and produces videos with improved quality. This is achieved by scaling the class embeddings and normalizing the outputs of the model. Consequently, we demonstrate that our proposed method can produce videos with more distinct class differences and improved quality.

## 3. Method

**Figure 2:**
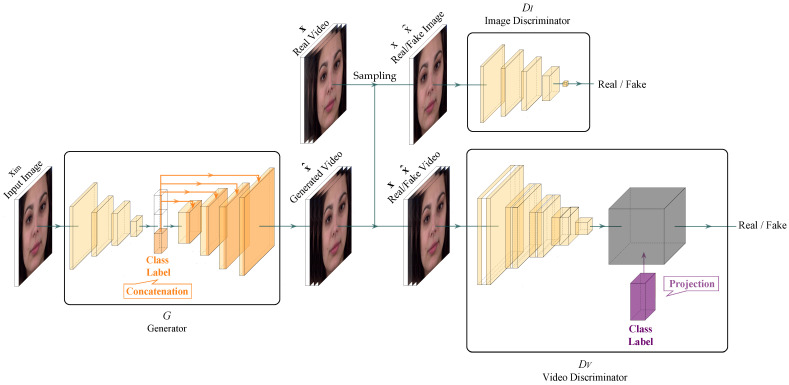
GammaGAN architecture which is visualised using PlotNeuralNet [50]. Our proposed model consists of three networks: generator *G*, image discriminator $D_{I}$, and video discriminator $D_{V}$. We adopt ImaGINator [33] as our backbone, sharing the same generator and image discriminator. The utilization of class labels varies between networks: the generator *G* uses the concatenation method [11] (orange), whereas the video discriminator $D_{V}$ employs the projection method [12] (purple). The details of our proposed method are encapsulated within the black box in the video discriminator $D_{V}$, which are described in detail in Figure 3.

In this section, we propose GammaGAN, an enhanced video discriminator network, featuring two novel techniques: scaling class embeddings and normalizing outputs. The proposed GammaGAN architecture, as illustrated in Figure 2, consists of a generator *G*, an image discriminator $D_{I}$, and a video discriminator $D_{V}$. The generator *G* uses a single image, noise, and a class label to generate a realistic video. The image discriminator $D_{I}$ randomly selects a frame from either a real or fake video and determines the appearance of the sample rather than its motion. Given a video and class label, the video discriminator $D_{V}$ determines whether the motion in the video is appropriate.

In particular, the methods used to inject the class label differed among the networks. Class labels are injected into the feature map in generator *G* by concatenation. In contrast, the video discriminator $D_{V}$ uses the proposed projection method to inject a class label, as shown in Figure 2.

We employed the ImaGINator architecture [33], which shares the same generator and image discriminator as our network, as a backbone to evaluate the effectiveness of our video discriminator. The ImaGINator network uses only the concatenation method to inject class information into networks.

In the following sections, we introduce details of GammaGAN by providing mathematical descriptions of our method and explaining its application to our model. In addition, we describe the objective function of the training process.

### 3.1. GammaGAN Video Discriminator

#### 3.1.1. Mathematical Description

Our goal is to generate a realistic video from a single image and a class label while preserving the class differences and enhancing the quality of the video. To this end, we propose an enhanced projection method for conditional video generation, GammaGAN. Rather than using conventional concatenation [11] to inject a class label into the discriminator, we apply our new approach based on the projection method [12].

The projection discriminator [12] is an alternative to the concatenation method [11,13,14,17,18] for injecting class information into the discriminator. The operation of the projection discriminator is based on two fundamental principles. First, it uses embeddings to train the model, specifically class embeddings and feature vectors. Second, it calculates the inner products of the class embeddings and feature vectors. This allows the network to understand the relationships and similarities between them. Therefore, the projection discriminator leverages class information more effectively than the concatenation methods.

Discriminators in cGANs learn to distinguish between real and fake samples from each distribution, given conditioning information: a class label [1,11]. The objective function of the cGANs’ discriminators [11,12,29] can be written as:

(1) $$\begin{array}{lll} L(D) & = & -E_{y∼p(y)}\left[E_{x∼p(x|y)}\left[\log(D(x,y))\right]\right]\\ & & -E_{y∼q(y)}\left[E_{x∼q(x|y)}\left[\log(1-D(x,y))\right]\right]\\ & = & -\int \log(D(x,y))p(x,y)\;dx\;dy\\ & & -\int \log(1-D(x,y))q(x,y)\;dx\;dy, \end{array}$$

where x∈X represents the input data and y={1,…,c}∈Y is a class label (class information). p(y) and q(y) are the true and fake label marginal distributions, respectively. p(x|y) and q(x|y) are the true and fake data distributions conditional on *y*, respectively. p(x,y) and q(x,y) are the true and fake joint distributions of x and *y*, respectively [29].

Gamma-exponentiated conditional probability. To propose our method, we denote x as the input vector and y as class information (i.e., label; we assume that the label y is a one-hot vector). In cGANs’ adversarial loss (1), D(x,y) represents the probability that the discriminator estimates the pair of input data x and class label y as real. The joint probability can be expressed using the conditional probability and the marginal probability,

(2) $$\begin{array}{c} D(x,y)=D(y|x)D(x)=A(f(x,y)), \end{array}$$

where A is the activation function. A sigmoid function (logistic function) is applied to our task. In this context, f(x,y) denotes the logit, which is the output value generated by the model before the activation function is applied.

Our goal is to increase class differences during training. To formulate our idea, we define $D_{\gamma }$ by exponentiating the conditional probability as follows:

(3) $$\begin{array}{c} D_{\gamma }\left(x,y\right):={\left(D\left(y|x\right)\right)}^{\gamma }D\left(x\right)=A\left(f_{\gamma }\left(x,y\right)\right), \end{array}$$

where γ is a real number used to weigh the importance of the conditional information in our model.

To express the logit itself, we can take the inverse function of the activation function on both sides of the equation as follows:

(4) $$\begin{array}{c} f_{\gamma }\left(x,y\right)=A^{-1}\left(D_{\gamma }\left(x,y\right)\right). \end{array}$$

Now, (4) can simply be expanded by the definition of logit as follows:

(5) $$\begin{array}{cc} f_{\gamma }\left(x,y\right)\; & =A^{-1}\left(D_{\gamma }\left(x,y\right)\right)\\ \; & =A^{-1}\left({\left(D(y|x)\right)}^{\gamma }D(x)\right)\\ \; & =\log\left(\frac{p{\left(y|x\right)}^{\gamma }p\left(x\right)}{q{\left(y|x\right)}^{\gamma }q\left(x\right)}\right)\\ \; & =\log\left(\frac{p{\left(y|x\right)}^{\gamma }}{q{\left(y|x\right)}^{\gamma }}\right)+\log\left(\frac{p(x)}{q(x)}\right)\\ \; & =\gamma \left(\underset{\mathrm{Label}\;\mathrm{Matching}}{\underbrace{\log\left(\frac{p(y|x)}{q(y|x)}\right)}}\right)+\left(\underset{\mathrm{Marginal}\;\mathrm{Matching}}{\underbrace{\log\left(\frac{p(x)}{q(x)}\right)}}\right)\\ \; & :=r(y|x)+r(x), \end{array}$$

where r(y|x) and r(x) are the log-likelihood ratios, respectively [12,20].

Gamma-scaled class embeddings. Our method can be achieved by weighting the class embeddings to increase class differences. First, the logit of the projection discriminator can be derived as follows [12]:

(6) $$\begin{array}{c} f\left(x,y;\theta \right)=y^{T}V\phi \left(x;\theta_{\Phi }\right)+\psi \left(\phi \left(x;\theta_{\Phi }\right);\theta_{\Psi }\right), \end{array}$$

where *V* is the embedding matrix, which includes the embedding vectors for all the classes, $\phi (\cdot ,\theta_{\Phi })$ is the feature vector of x, and $\psi (\cdot ,\theta_{\Psi })$ is a scalar function that is denoted for the normalization constant [12]. Equation (6) can be written when y=c as follows:

(7) $$\begin{array}{c} f\left(x,y=c\right)=v_{c}^{T}\phi \left(x\right)+\psi \left(\phi \left(x\right)\right), \end{array}$$

where $v_{c}^{T}$ is the class embedding of y [12].

Now, we can derive the proposed logit in (5) using (7),

(8) $$\begin{array}{cc} f_{\gamma }\left(x,y=c\right) & :=\gamma \left(v_{c}^{T}\phi \left(x\right)\right)+\psi \left(\phi \left(x\right)\right)\\ & =\left(\gamma v_{c}^{T}\right)\phi \left(x\right)+\psi \left(\phi \left(x\right)\right), \end{array}$$

where γ represents the weight of the class embedding of y=c, demonstrating that our method can increase the class differences by simply scaling the class embeddings. Note that the scale γ is a learnable parameter.

Normalized outputs. We introduce the normalization technique in GammaGAN. This constrains output (logit) growth, which can occur when the learnable parameter γ diverges, by normalizing the two terms. In addition, this allows our network to be trained by balancing the importance of the two terms and observing their relative significance. Our proposed method, GammaGAN, can be expressed as follows:

(9) $$\begin{array}{cc} f_{\gamma }\left(x,y=c\right) & :=\frac{\gamma }{\gamma +1}\left(v_{c}^{T}\phi \left(x\right)\right)+\frac{1}{\gamma +1}\left(\psi \left(\phi \left(x\right)\right)\right)\\ & =\frac{1}{\gamma +1}\left(\left(\gamma v_{c}^{T}\right)\phi \left(x\right)+\psi \left(\phi \left(x\right)\right)\right). \end{array}$$

Furthermore, (9) can be generalized because it represents the specific case when y=c. By generalizing (9), we derive our method as follows:

(10) $$\begin{array}{cc} f_{\gamma }\left(x,y\right) & =\frac{\gamma }{\gamma +1}\left(y^{T}V\phi \left(x\right)\right)+\frac{1}{\gamma +1}\left(\psi \left(\phi \left(x\right)\right)\right)\\ & =\frac{1}{\gamma +1}\left(\left(\gamma y^{T}V\right)\phi \left(x\right)+\psi \left(\phi \left(x\right)\right)\right), \end{array}$$

where *V* is the class embedding matrix.

#### 3.1.2. Architecture

**Figure 3:**
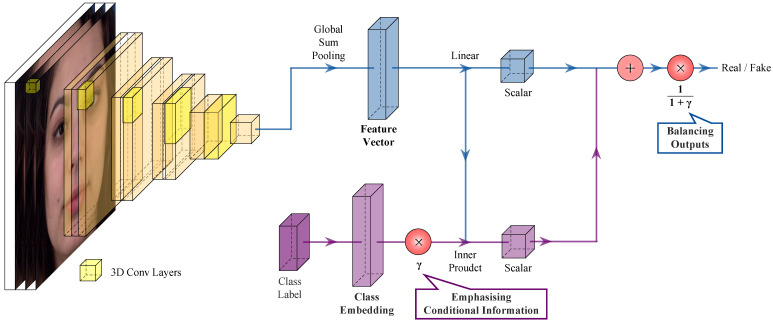
Proposed video discriminator. Our proposed model, the video discriminator in GammaGAN, consists of two streams: the data stream (blue) and the class embedding stream (purple). First, the data stream is designed to extract feature vectors and normalize the outputs for balance using $\frac{1}{\gamma +1}$, leading to improved video quality. Second, the class embedding stream utilizes a novel learnable parameter, γ, to generate scaled class embeddings. These scaled embeddings emphasize class information, enabling our model to generate more distinguishable videos between classes.

Figure 3 illustrates our proposed video discriminator, which is composed of two streams: the data stream (blue) and the class embedding stream (purple). The first and second terms in (10) represent the class embedding stream and the data stream, respectively.

First, the data stream aims to extract valuable feature vectors from the video data using 3D convolutional layers. Once the feature vectors are obtained, the data stream splits into two branches: one continuing as the data stream for unconditional marginal matching and the other feeding into the class embedding stream for conditional label matching. The branch that continues as the data stream transforms the feature vectors into a scalar using a linear layer, whereas the branch directed into the class embedding stream uses feature vectors to perform the inner product with class embeddings.

Second, the class embedding stream focuses on obtaining class embeddings and emphasizing the class information. The one-hot encoded class labels transform their dimensions through an embedding layer, which enables the class embeddings to perform inner products with the feature vectors obtained from the data stream. The class information is emphasized through multiplication by our proposed constant, denoted as γ. After obtaining the inner product, resulting in a scalar, the scalar is added to the scalar from the data stream. Subsequently, the scalars from both the data and class embedding streams are balanced using our proposed method, denoted as $\frac{1}{\gamma +1}$. It is crucial to note that the normalization technique gains its significance only when the class embeddings are scaled, given that the scaling factor γ of class embeddings is a learnable parameter.

Therefore, the data stream is designed to extract feature vectors and normalize outputs; the model can balance outputs, resulting in improved video quality. On the other hand, the class embedding stream is designed to obtain class embeddings and scale these class embeddings. Class information can be emphasized by scaling class embeddings, enabling the model to generate more distinguishable videos with different classes.

### 3.2. Objective Function

In this section, the objective function of the training process is introduced. First, we describe the full objective function, followed by the objective functions of the generator *G*, the image discriminator $D_{I}$, and the video discriminator $D_{V}$. Since we use ImaGINator [33] as our backbone, the losses for the generator and discriminators are similar to those of the ImaGINator.

#### 3.2.1. Full Objective Function

The objective function for training our model is described in the following way. Note that real images and videos are denoted by *x* and x, respectively, and fake images and videos are denoted by $\hat{x}$ and $\hat{x}$. Since we aim to generate a video from noise *z*, an input image $x_{im}$, and a class label y, the process can be represented as follows:

(11) $$\begin{array}{c} G:\left\{z,x_{im},y\right\}\to \hat{x}, \end{array}$$

where $\hat{x}$ represents the generated video output.

Generator *G* aims to generate a realistic video using noise *z*, an input image $x_{im}$, and a class label y. In contrast, the video discriminator $D_{V}$ differentiates between real videos x and fake videos $\hat{x}$. Similarly, the image discriminator $D_{I}$ distinguishes between sampled frames from real videos and fake videos (*x* and $\hat{x}$). The objective function during training is defined as follows:

(12) $$\begin{array}{c} \arg\min_{G}\max_{D_{I},D_{V}}L\left(G,D_{I},D_{V}\right). \end{array}$$

We introduce our full objective function, which consists of two losses: the adversarial loss $L_{adv}$ and the reconstruction loss $L_{rec}$,

(13) $$\begin{array}{c} L\left(G,D_{I},D_{V}\right)=L_{adv}\left(G,D_{I},D_{V}\right)+\lambda L_{rec}\left(G\right), \end{array}$$

where the parameter λ is used to stabilize and ensure the balance between these two losses ($L_{adv}$ and $L_{rec}$) during the training [33]. In the next step, the objective functions of the generator *G*, the image discriminator $D_{I}$, and the video discriminator $D_{V}$ are described, in that order.

#### 3.2.2. Generator Loss

The objective function of generator *G* consists of adversarial loss and reconstruction loss with λ,

(14) $$\begin{array}{c} L_{G}=L_{adv}+\lambda L_{rec}. \end{array}$$

The adversarial loss of the generator is defined as follows:

(15) $$\begin{array}{c} L_{\mathrm{adv}}=L_{adv}^{I}+L_{adv}^{V}, \end{array}$$

where $L_{adv}^{I}$ and $L_{adv}^{V}$ are the image adversarial loss and the video adversarial loss, respectively. The full adversarial loss $L_{\mathrm{adv}}$ is the sum of the image adversarial loss $L_{adv}^{I}$ and the video adversarial loss $L_{adv}^{V}$.

The image adversarial loss is defined as:

(16) $$\begin{array}{cc} L_{adv}^{I} & =E_{z∼p_{z}\left(z\right),x_{im},y}\left[1-\log D_{I}\left(\hat{x}\right)\right]\\ \; & =E_{z∼p_{z}\left(z\right),x_{im},y}\left[1-\log D_{I}{\left(G\left(z,x_{im},y\right)\right)}^{\prime }\right], \end{array}$$

where the generator attempts to minimize $L_{adv}^{I}$ by penalizing the distance between the distribution of the generated image samples $\hat{x}$ and real image samples *x* [41]. $\hat{x}=G{\left(z,x_{im},y\right)}^{\prime }$ denotes a random image sampled from the generated video, $\hat{x}=G\left(z,x_{im},y\right)$.

The video adversarial loss is similarly defined as:

(17) $$\begin{array}{cc} L_{adv}^{V} & =E_{z∼p_{z}\left(z\right),x_{im},y}\left[1-\log D_{V}\left(\hat{x},y\right)\right]\\ \; & =E_{z∼p_{z}\left(z\right),x_{im},y}\left[1-\log D_{V}\left(G\left(z,x_{im},y\right),y\right)\right], \end{array}$$

where the generator attempts to minimize $L_{adv}^{V}$ by penalizing the distance between the distribution of generated video samples $\hat{x}$ and real video samples x [41].

The reconstruction loss is utilized for the generated video’s coherence and authenticity, which allows the generator to generate realistic and plausible videos:

(18) $$\begin{array}{c} L_{\mathrm{rec}}=E\left[{\left|x-\hat{x}\right|}_{1}\right]=E\left[{\left|x-G(z,x_{im},y)\right|}_{1}\right], \end{array}$$

where x is the real video, and $\hat{x}=G\left(z,x_{im},y\right)$ is the generated video from the generator [33].

#### 3.2.3. Image Discriminator Loss

The image discriminator $D_{I}$ learns to distinguish whether a sampled image (*x* or $\hat{x}$) is from the real distribution or the fake distribution, which attempts to maximize the image discriminator loss. The loss function of the image discriminator is expressed as follows:

(19) $$\begin{array}{cc} L_{I} & =E_{x∼p_{\mathrm{data}}}\left[\log D_{I}\left(x\right)\right]\\ \; & +E_{z∼p_{z}\left(z\right),x_{im},y}\left[1-\log D_{I}\left(\hat{x}\right)\right]\\ \; & =E_{x∼p_{\mathrm{data}}}\left[\log D_{I}\left(x\right)\right]\\ \; & +E_{z∼p_{z}\left(z\right),x_{im},y}\left[1-\log D_{I}{\left(G\left(z,x_{im},y\right)\right)}^{\prime }\right], \end{array}$$

where *x* and $\hat{x}$ represent a randomly sampled frame from real videos x and generated videos $\hat{x}$, respectively, for the image discriminator [33].

#### 3.2.4. Video Discriminator Loss

Similarly, the video discriminator $D_{V}$ learns to determine whether a sampled video (x or $\hat{x}$) is from the real distribution or the fake distribution, which attempts to maximize the video discriminator loss. The loss function of the video discriminator is as follows:

(20) $$\begin{array}{cc} L_{V} & =E_{x∼p_{\mathrm{data}},y}\left[\log D_{V}\left(x,y\right)\right]\\ \; & +E_{z∼p_{z}\left(z\right),x_{im},y}\left[1-\log D_{V}\left(\hat{x},y\right)\right]\\ \; & =E_{x∼p_{\mathrm{data}},y}\left[\log D_{V}\left(x,y\right)\right]\\ \; & +E_{z∼p_{z}\left(z\right),x_{im},y}\left[1-\log D_{V}\left(G\left(z,x_{im},y\right),y\right)\right], \end{array}$$

where x represents the real video, and *z* is the latent variable, which signifies noise [33].

## 4. Experimental Results

In this section, we present our experimental results and quantitatively and qualitatively evaluate the proposed method.

### 4.1. Experimental Setup

#### 4.1.1. Dataset

Our experiments utilized the MUG facial expression database provided by the Multimedia Understanding Group [30]. MUG is a facial expression dataset that consists of seven labels: happiness, sadness, surprise, anger, disgust, fear, and neutral. For the experiment, we used six labels corresponding to happiness, sadness, surprise, anger, disgust, and fear. The neutral expression was used as the initial frame to generate videos. Each video has a resolution of 896 × 896 pixels and contains between 50 and 160 frames. There are 931 videos and 52 subjects in total. The intensity of facial expressions varies from frame to frame. Each video initially starts with a neutral facial expression, progresses to the most expressive point around half of the frames, and returns to the neutral facial expression again.

#### 4.1.2. Implementation Details

We conducted end-to-end training on a single A100 NVIDIA GPU using PyTorch [51]. We utilized ImaGINator [33] as our backbone, sharing the same generator and image discriminator architecture. Our experiment focused on evaluating the effectiveness of the proposed video discriminator. For comparison, we selected approximately 32 frames from the first half of each video because the videos typically reached the peak of facial expression intensity around their midpoints. These frames progressively increased the intensity of the facial expressions from neutral to the maximum. To ensure a fair comparison, we used the Adam optimizer [52] with parameters $\beta_{1}=0.5$ and $\beta_{2}=0.999$ for all three networks: the generator, the image discriminator, and the video discriminator, matching the values used in ImaGINator [33]. We set the learning rate for all parameters, including the proposed weight parameter γ, to $2\times 10^{-4}$, with the exception of λ, which was set to $1\times 10^{-4}$. The λ was used to balance the adversarial and reconstruction losses, matching the value used in our backbone architecture. The batch size was set to 64 during training.

#### 4.1.3. Evaluation Metrics

For qualitative evaluation, we compared the generated videos with the ground truth using three metrics: PSNR, SSIM, and LPIPS.

The Peak Signal-to-Noise Ratio (PSNR) measures image quality based on pixel-level differences. A higher PSNR value indicates better quality of the generated images.

The Structural Similarity Index Measure (SSIM) [53] measures the structural similarity aligned with human perception. A higher SSIM value indicates better image quality.

The Learned Perceptual Image Patch Similarity (LPIPS) [54] is a novel metric that provides a more perceptual evaluation between two images compared to other metrics. LPIPS calculates the similarities between feature vectors extracted from a pre-trained VGG [55] network, enabling more perceptual similarity evaluation between the two images. A lower LPIPS value indicates better image quality.

#### 4.1.4. Evaluation Method

To ensure fairness and precision, we applied our evaluation method to the pre-trained ImaGINator model [33], which Y. Wang et al. shared. We chose 10 subjects not part of the training set out of the 52 for video generation. The neutral facial expressions of these 10 subjects were utilized as the input for the generator, producing six unique facial expressions for each subject. After the videos were generated, they were compared frame-by-frame with the ground truth corresponding to each facial expression.

### 4.2. Ablation Study

In this section, we present the results of our ablation study, evaluating the essential elements of our proposed method, scaling class embeddings, and normalizing the outputs. We compared the performance of four different models according to their use of projection, scaling, and normalization, as shown in Table 1. First, we applied the projection method [12] to our conditional video generation task, assessing its effectiveness in the video domain. Second, we evaluated our method without the normalization technique to scrutinize the impact of scaled class embeddings. Lastly, we assessed our proposed method, GammaGAN, to demonstrate its enhanced performance.

#### 4.2.1. Effectiveness of Normalization

GammaGAN employs the normalization technique as outlined in (10), balancing the outputs of the two streams: the data stream and the class embedding stream. An ablation study was conducted to evaluate the effectiveness of the normalization technique in our proposed method, GammaGAN, both qualitatively and quantitatively.

**Figure 4:**
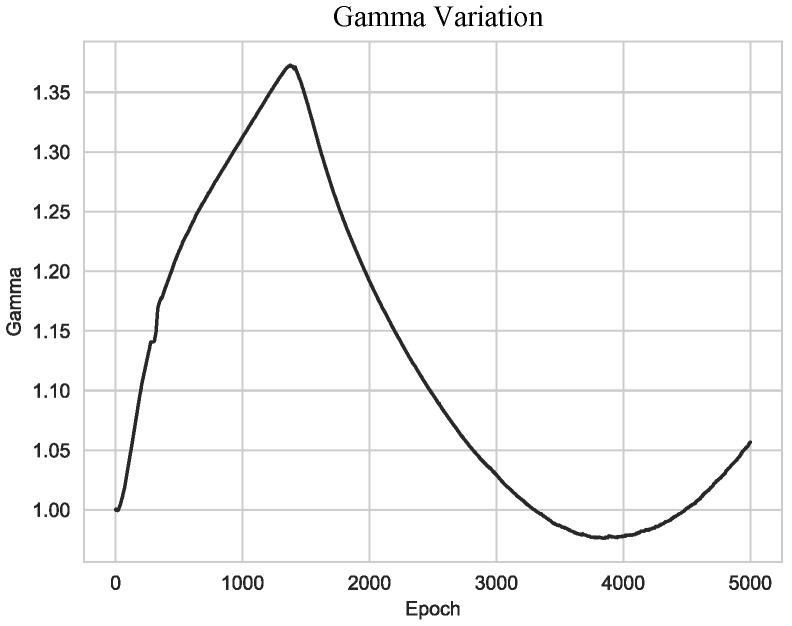
Gamma variation during training. The graph shows the variation of the proposed learnable parameter, γ, during training from epoch 0 to epoch 5000.

To begin with, Figure 4 represents the variations in the weight parameter γ throughout training when γ is initialized with a value of 1.0. The observed fluctuation in the weight parameter γ demonstrates that our method adaptively and effectively balances the data stream and the class embedding stream. This suggests that our model automatically learns what elements to give more attention to between feature vectors and class embeddings during training.

**Figure 5:**
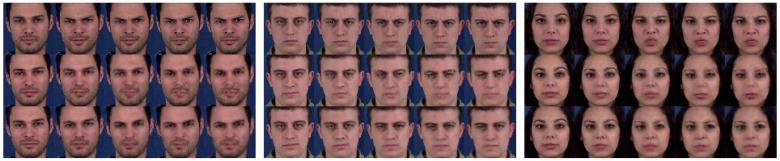
Effectiveness of normalization. An ablation study was conducted to demonstrate the effects of normalization. All the results presented have the label ‘anger’. The first row represents the ground truth. The second row represents the results without the normalization technique in GammaGAN, yielding lower-quality videos, whereas the third row represents the improved results of GammaGAN with normalization.

For qualitative comparison, Figure 5 illustrates the effectiveness of our normalization technique by comparing GammaGAN with and without the normalization method. The second row, without normalization, and the third row, representing GammaGAN with normalization, clearly show differences in video quality, indicating that GammaGAN generates improvement in videos when using normalization. This suggests that our normalization technique enables the model to automatically learn to balance feature vectors in the data stream and class embeddings in the class embedding stream, giving adaptive attention to feature vectors during training.

A quantitative comparison was conducted to evaluate the performance of the proposed method, as presented in Table 1. Our proposed method, GammaGAN with the normalization method, performed better than GammaGAN without the normalization method. This demonstrates that our normalization method can effectively enhance the quality of videos generated by the model.

#### 4.2.2. Effectiveness of Scaling Class Embeddings

In this section, we discuss the effectiveness of scaling class embeddings in our proposed method, GammaGAN. As shown in Table 1, our method without normalization yielded results inferior to those of Proj-GAN [12], suggesting that the utilization of both scaling and normalization methods is essential for optimal performance.

**Figure 6:**
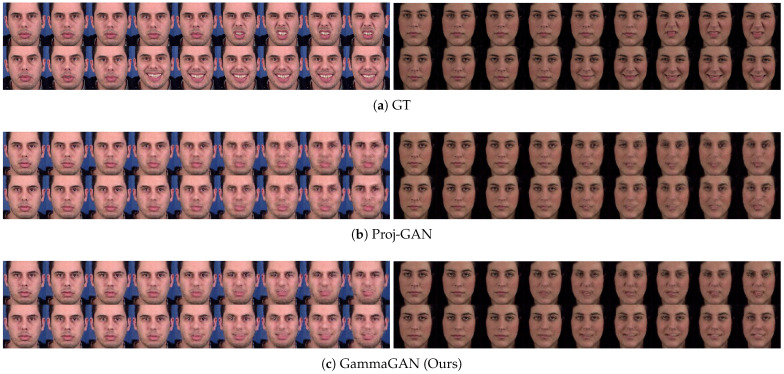
Comparison between Proj-GAN and GammaGAN. (**a**) represents the ground truth of generated videos. (**b**) represents the generated videos when Proj-GAN [12] is applied to the video discriminator. (**c**) shows the results of GammaGAN. The first rows represent ‘disgust’ (**top**), and the second rows represent ‘happiness’ (**bottom**) in each of (**a**–**c**).

We conducted an experiment comparing our method, GammaGAN, with Proj-GAN [12] to demonstrate the effectiveness of scaling class embeddings and the normalization technique, factors that differentiate it from Proj-GAN. As illustrated in Figure 6, our method produces videos that more effectively distinguish between classes than those generated by Proj-GAN, particularly between the labels ‘disgust’ and ‘happiness’. These results demonstrate that our proposed method effectively differentiates between different classes when class embeddings are emphasized by our proposed γ, and that our method generates enhanced video quality simultaneously using the normalization technique.

For the quantitative comparison, our proposed method, GammaGAN with a normalization technique, outperformed other experiments in terms of PSNR and SSIM, as shown in Table 1. However, our method underperforms in the LPIPS metric. A lower LPIPS score is observed with the model when only the projection method is applied. This stems from our training emphasis on improving PSNR and SSIM metrics without including additional loss. Even though the LPIPS metric seems better with the projection method, qualitative evaluations in Figure 6 highlight the efficacy of our method, specifically in distinguishing class-specific differences and improving video quality. This demonstrates the effectiveness of our proposed method, particularly when using the two essential techniques: scaling and normalization. As a result, GammaGAN generates more realistic videos by effectively differentiating facial expressions between various classes, achieved by scaling class embeddings. In addition, our method enhances video quality by balancing feature vectors and class embeddings during training.

### 4.3. Comparative Results

#### 4.3.1. Quantitative Evaluation

For quantitative comparison, we compared the results of the proposed GammaGAN with those of VGAN [31], MoCoGAN [32], and ImaGINator [33]. The results are presented in Table 2. Our method led to relative improvements of 1.61%, 1.66%, and 0.36% in terms of PSNR, SSIM, and LPIPS, respectively, compared with ImaGINator [33] on the MUG facial expression dataset [30]. These improvements indicate that our method enhances the quality of videos by scaling class embeddings using our proposed learnable constant, γ, and normalizing the outputs.

#### 4.3.2. Qualitative Evaluation

**Figure 7:**
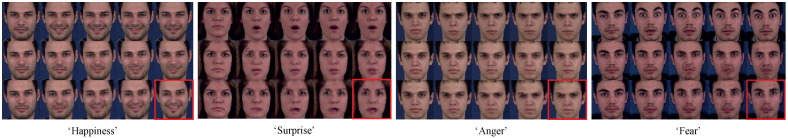
Comparative results between ImaGINator and GammaGAN. Each figure represents the output video results for the labels ‘happiness’, ‘surprise’, ‘anger’, and ‘fear’. Within each figure, the first row represents the ground truth, the second row shows the results of ImaGINator, and the third row shows the results from GammaGAN (Ours). The red boxes highlight the last frames in each video, where the intensity of facial expression is assumed to be at its peak. GammaGAN produces more plausible videos than the previous method, ImaGINator [33].

Figure 7 shows the qualitative results of our method, GammaGAN, compared with a previous video generation method, ImaGINator [33]. As seen in Figure 7, our method produces more distinguishable videos between each class label. This distinction is due to the different utilization of class labels and our proposed method. Whereas ImaGINator simply concatenates a class label in the video discriminator, our method, GammaGAN, employs the class label not only in a projection method but also in assigning γ weights to the class embeddings.

**Figure 8:**
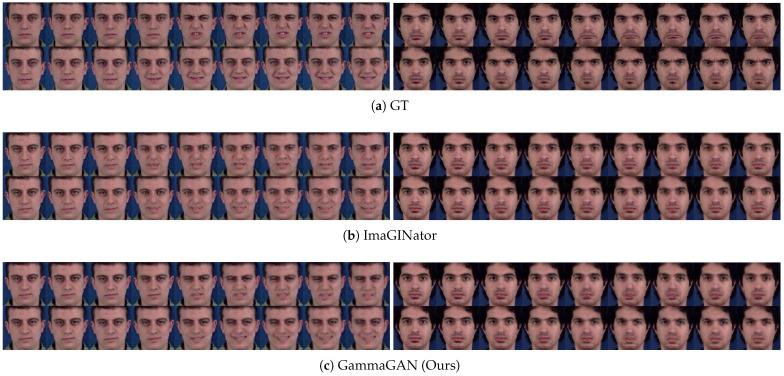
Comparison between different classes using ImaGINator and GammaGAN. (**a**) represents the ground truth, (**b**) represents the results of ImaGINator [33], and (**c**) represents the results of GammaGAN. Each result in (**a**–**c**) illustrates generated videos labeled as ‘disgust’ (**top left**), ‘happiness’ (**bottom left**), ‘sadness’ (**top right**), and ‘anger’ (**bottom right**). GammaGAN generates more distinctive video results between different classes.

Figure 8 demonstrates the enhanced ability of the proposed method to distinguish between different facial expression classes. Whereas distinctions between ‘disgust’ and ‘happiness’ and between ‘sadness’ and ‘anger’ from the previous method are somewhat subtle, there is an improvement in the differentiation of classes when our method is applied. This improvement is due to the enhancement of the class embeddings by the proposed method. In addition, as shown in Figure 8, our method improves the quality of frames and videos, resulting in fewer artifacts compared with the previous method because of our normalization technique.

## 5. Conclusions

We introduced GammaGAN, a novel network designed for conditional video generation using a single image and a class label. Our method successfully increased the differences between classes and enhanced the quality of the generated videos using two essential methods: scaling class embeddings and normalizing outputs. Our approach enhances the differences between classes by scaling class embeddings using a learnable parameter, γ, effectively emphasizing conditional information. Furthermore, our model balances the data stream and the class embedding stream by normalizing the outputs, leading to improved quality of videos. This suggests that our approach has the potential for further advancement in conditional video generation. Nonetheless, our method has shown a limitation, particularly with the LPIPS metric. One of the suggestions is to include additional loss terms to improve the performance of our model. This should be done in future work.

## Figures and Tables

**Table 1:** Ablation study on GammaGAN. The results from our models are shown in bold, and the best-performing models are underlined.

Model	Method			Metric		
	**Projection**	**Scaling**	**Normalization**	**PSNR**↑	**SSIM**↑	**LPIPS**↓
ImaGINator [33] ^1^				24.8779	0.8214	0.1119
Proj-GAN [12]	✓			25.2851	0.8344	0.1070
GammaGAN w/o normalization	✓	✓		25.1176	0.8288	0.1179
**GammaGAN w/normalization (Ours)**	✓	✓	✓	**25.2917**	**0.8346**	**0.1112**

^1^ We re-evaluated the pre-trained ImaGINator.

**Table 2:** Comparison of PSNR, SSIM, and LPIPS among different video generation methods. The results from our models are shown in bold, and the best-performing models are underlined.

Method	PSNR ↑	SSIM ↑	LPIPS ↓
VGAN [31]	14.54	0.28	-
MoCoGAN [32]	18.16	0.58	-
ImaGINator [33]	22.63	0.75	-
ImaGINator [33] ^1^	24.8779	0.8214	0.1119
**GammaGAN (Ours)**	**25.2917**	**0.8346**	**0.1112**

^1^ We re-evaluated the pre-trained ImaGINator [33] with our evaluation method.

## Data Availability

Data sharing not applicable.

## Abbreviations

The following abbreviations are used in this manuscript:

GANs	Generative Adversarial Networks
cGANs	Conditional Generative Adversarial Networks
MUG	Multimedia Understanding Group
VGG	Visual Geometry Group
PSNR	Peak Signal-to-Noise Ratio
SSIM	Structural Similarity Index Measure
LPIPS	Learned Perceptual Image Patch Similarity
